# Apnoeic oxygenation in morbid obesity: a randomised controlled trial comparing facemask and high-flow nasal oxygen delivery

**DOI:** 10.1016/j.bja.2021.12.011

**Published:** 2022-01-11

**Authors:** John Schutzer-Weissmann, Thomas Wojcikiewicz, Anil Karmali, Asta Lukosiute, Ruoyi Sun, Rafiq Kanji, Ahmed R. Ahmed, Sanjay Purkayastha, Stephen J. Brett, Jonathan Cousins

**Affiliations:** 1 Imperial College Healthcare NHS Trust, London, UK; 2 The Royal Marsden Hospital NHS Foundation Trust, London, UK; 3 Royal Surrey NHS Foundation Trust, Guildford, UK; 4 London North West University Healthcare NHS Trust, Harrow, UK; 5 Guy's and St Thomas' NHS Foundation Trust, London, UK; 6 Department of Surgery and Cancer, Imperial College London, UK

**Keywords:** apnoeic oxygenation, apnoeic ventilation, bariatric anaesthesia, desaturation risk, high-flow nasal oxygen, obesity, safe apnoea time, THRIVE

## Abstract

**Background:**

Obesity is a risk factor for airway-related incidents during anaesthesia. High-flow nasal oxygen has been advocated to improve safety in high-risk groups, but its effectiveness in the obese population is uncertain. This study compared the effect of high-flow nasal oxygen and low-flow facemask oxygen delivery on duration of apnoea in morbidly obese patients.

**Methods:**

Morbidly obese patients undergoing bariatric surgery were randomly allocated to receive either high-flow nasal (70 L min^−1^) or facemask (15 L min^−1^) oxygen. After induction of anaesthesia, the patients were apnoeic for 18 min or until peripheral oxygen saturation decreased to 92%.

**Results:**

Eighty patients were studied (41 High-Flow Nasal Oxygen, 39 Facemask). The median apnoea time was 18 min in both the High-Flow Nasal Oxygen (IQR 18–18 min) and the Facemask (inter-quartile range [IQR], 4.1–18 min) groups. Five patients in the High-Flow Nasal Oxygen group and 14 patients in the Facemask group desaturated to 92% within 18 min. The risk of desaturation was significantly lower in the High-Flow Nasal Oxygen group (hazard ratio=0.27; 95% confidence interval [CI], 0.11–0.65; *P*=0.007).

**Conclusions:**

In experienced hands, apnoeic oxygenation is possible in morbidly obese patients, and oxygen desaturation did not occur for 18 min in the majority of patients, whether oxygen delivery was high-flow nasal or low-flow facemask. High-flow nasal oxygen may reduce desaturation risk compared with facemask oxygen. Desaturation risk is a more clinically relevant outcome than duration of apnoea. Individual physiological factors are likely to be the primary determinant of risk rather than method of oxygen delivery.

**Clinical trial registration:**

NCT03428256.

**Editor's key points**

• High-flow nasal oxygenation is useful to prevent hypoxaemia during attempts at securing the airway after induction of anaesthesia, but its efficacy in morbidly obese patients is not clear.
• Compared with morbidly obese patients receiving low-flow oxygenation via a facemask, those receiving high-flow nasal oxygenation were less likely to become hypoxaemic during 18 min of apnoea after induction of anaesthesia.
• Morbid obesity did not preclude apnoeic oxygenation. However, future studies need to establish the role of high-flow nasal oxygenation during apnoea in morbidly obese patients, as 5 of 41 patients became hypoxaemic.

There has been a resurgence in interest in apnoeic oxygenation techniques since the advent of high-flow heated and humidified oxygen delivery systems.^1^ Several studies have investigated the influence of oxygen delivery characteristics, notably flow rate and the proximity of fresh gas flow to the respiratory epithelium, on apnoeic oxygenation and ventilation. Broadly, the conclusions have been that these processes are more efficient with higher flow rates of oxygen delivered closer to the lung.^2^ The evidence is limited, in particular by the use of surrogate markers of arterial gas tension such as end-tidal concentration. Whether high-flow nasal oxygen (HFNO) delivery promotes apnoeic ventilation remains a subject of debate.^3^

Obesity has been associated with failure of apnoeic oxygenation techniques.^4^ In the UK, more than a quarter of adults are obese and obesity is increasingly common worldwide.^5^ Obesity presents many challenges to the anaesthetist,^6^ and airway-related incidents are more common in obese patients.^7^^,^^8^ Understanding the factors that influence the efficiency of apnoeic oxygenation in obese patients might improve safety of airway management in this high-risk group. Landmark papers describing apnoeic oxygenation using HFNO at the time of airway surgery concluded that, although apnoea could be reliably extended in non-obese patients using this technique,^9^ the safe upper limit of apnoea in the presence of morbid obesity may be as low as 5 min but that this needs confirmation by an experimental human physiological study.^10^

In this physiological study, we explored the safe upper limit of apnoea in morbidly obese patients. We compared the effect of oxygen flow rate and proximity of fresh gas flow to the respiratory epithelium on the duration of apnoea. Our primary outcome was the time to arterial haemoglobin oxygen desaturation to 92%. In addition, we measured arterial oxygen and carbon dioxide tension during apnoea to determine whether higher oxygen flow rate promotes more efficient apnoeic oxygenation, ventilation, or both.

## Methods

This study was registered with ClinicalTrials.gov (NCT03428256), approved by the Bloomsbury Research and Ethics Committee (17/LO/0742) and was conducted in a tertiary centre between October 2018 and September 2019. Two experienced bariatric anaesthetists and an operating department practitioner were present at all times. Emergency equipment and drugs were immediately available.

Eligible participants were recruited during a bariatric surgical clinic. Inclusion criteria were patients aged 18–65 yr with BMI >40 kg m^−2^. Exclusion criteria were inability to give informed consent; significant cardiac, peripheral vascular or respiratory disease; nasal obstruction; and predicted difficult facemask ventilation or intubation. Written, informed consent was obtained.

A secure online service (www.sealedenvelope.com) was used to randomise participants to HFNO or facemask oxygen (FM) groups. Minimisation ensured participants with diagnosed obstructive sleep apnoea using continuous positive airway pressure (CPAP) therapy were balanced between groups.

Participants were positioned at 45° mid-thoracic incline.^6^ In addition to standard monitoring,^11^ bispectral index (BIS™; Medtronic Limited, Boulder, CO, USA) and invasive arterial blood pressure were monitored throughout the study period.

In both groups, preoxygenation was provided for 3 min. Participants were asked to take vital capacity breaths during the third minute. In both groups, oxygen was delivered during preoxygenation and throughout apnoea.

In the FM group, oxygen was delivered via a tightly fitted anaesthetic facemask connected to a pressure-free circle circuit with 15 L min^−1^ oxygen.

In the HFNO group, oxygen was delivered via Optiflow™ (Fisher & Paykel Healthcare Limited, Auckland, New Zealand). During preoxygenation, flow was 35 L min^−1^ for the first minute, 50–70 L min^−1^ as tolerated for the next 2 min, and thereafter flow was maintained at 70 L min^−1^. Participants were instructed to keep their mouths closed throughout.

Anaesthesia was induced with fentanyl 2 μg kg^−1^ (predicted body weight [PBW]) and propofol infusion (Marsh model, plasma concentration target 6 μg ml^−1^). Rocuronium 1 mg kg^−1^ (PBW) was given after loss of verbal contact. An oropharyngeal airway and jaw-thrust manoeuvre were used to optimise airway patency until the study endpoint was reached. The ability to ventilate manually through a facemask was checked with a single insufflation in both groups – if not, the study was abandoned. Propofol infusion was titrated to BIS 40–60. Systolic blood pressure was maintained within 20% of baseline using metaraminol infusion with or without boluses. Further rocuronium doses were permitted at the anaesthetist's discretion if apnoea time exceeded 10 min to ensure optimum intubating conditions.

Onset of apnoea was defined as 1 min after rocuronium administration. Arterial blood gas samples were taken at baseline (before preoxygenation), at the end of preoxygenation, at the onset of apnoea (TA), and at 2, 4, 6, 9, 12, and 18 min (TA+2, 4, …, 18) min thereafter if arterial oxygen saturation (*S*ao_2_) remained >92%; or when *S*ao_2_ reached 92% if this occurred before TA+18. These samples were immediately refrigerated and processed sequentially at the end of the study (GEM Premier 4000; IL GmbH, Berlin, Germany).

When the study endpoint was reached (TA+18 or *S*ao_2_ 92%), a videolaryngoscope (McGrath™; Medtronic Limited, Watford, UK) was inserted and the trachea intubated.

The study was powered in respect of the primary outcome – time to desaturation to 92%. There were no extant data in obese patients given facemask oxygen during apnoea. In one study, using 5 L min^−1^ oxygen via nasopharyngeal catheter during apnoea in obese subjects, time to desaturation was 317 (standard deviation [sd], 80) s.^12^ We used this to estimate time to desaturation in the FM group. We estimated that time to desaturation in the HFNO group would be 20% longer. Allowing for up to 18 min of apnoea, more than double the expected apnoea time, we did not expect any censored events.

Using these estimates, 1:1 randomisation, two-tailed alpha 0.05, and 1-beta 0.9, a sample size of 35 per group was calculated to provide adequate power with respect to the primary outcome, but – given the uncertainty in these estimates – we chose a sample size of 40 per group.

Standard descriptive statistics were used to summarise the presenting features of two groups using mean and sd or median and inter-quartile range (IQR) and 95% confidence interval (95% CI). Time to desaturation is summarised using Kaplan–Meier curves and compared between groups using the log-rank statistic. Continuous data were checked for normality (d’Agostino and Pearson), and between-group differences were evaluated using either an independent *t*-test or Mann–Whitney *U*-test as appropriate. For those who desaturated during the 18 min period, the time to desaturation was compared between groups using the Mann–Whitney *U*-test. Arterial oxygen (*P*a_o2_) and carbon dioxide (*P*a_co2_) tension were compared between groups using independent *t*-tests. The Holm–Sidak method was used to correct for multiple comparisons. A two-tailed *P* value <0.05 was taken to indicate statistical significance.

Data were collated using Excel 2016 (Microsoft Corporation, Redmond, WA, USA) and analysed using Prism 8.3.0 (GraphPad Software Inc., San Diego, CA, USA). Data and documentation would be securely stored for 10 yr after the completion of the study.

## Results

**Fig 1:**
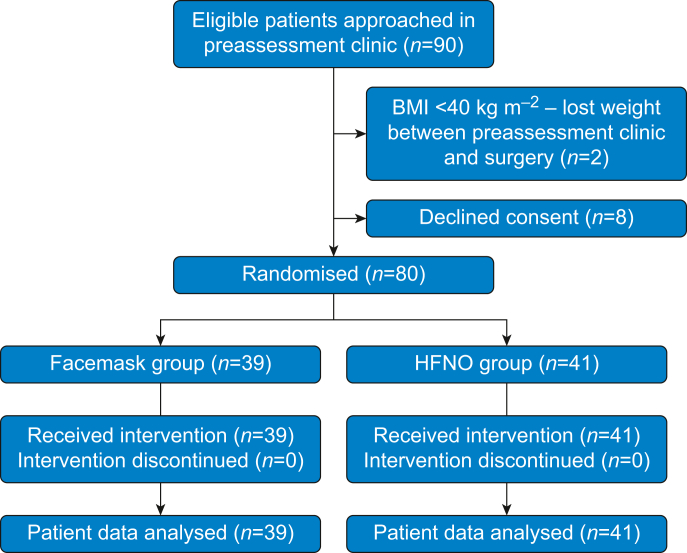
CONSORT diagram. CONSORT, Consolidated Standards of Reporting Trials; HFNO, high-flow nasal oxygen.

Eighty participants were randomised (41 patients for the HFNO group, 39 for the FM group). All completed the study (Fig. 1). No serious adverse events occurred. Ten participants in each group were using CPAP and baseline characteristics were similar in both groups (Table 1). Fifty-three patients received an additional rocuronium after 10 min of apnoea. Of the 60 patients who reached 18 min of apnoea without desaturation, 54 received an additional dose of rocuronium.

**Table 1:** Subject characteristics. Continuous variables are presented as median (IQR). CPAP was prescribed to patients diagnosed with obstructive sleep apnoea (OSA). STOP-Bang score is a validated tool to assess risk of OSA where a score ≥5 indicates a high risk of OSA.^38^ CPAP, continuous positive airway pressure; IQR, inter-quartile range; FM, facemask oxygen; HFNO, high-flow nasal oxygen.

	FM	HFNO
Participants	39	41
CPAP use	10	10
STOP-Bang score ≥5	10	13
Sex	31 F / 8 M	26 F / 15 M
Age (yr)	48 (38–54)	47 (36–55)
Weight (kg)	130 (122–139)	129 (118–144)
BMI (kg m^−2^)	46.7 (44.4–49.5)	46.6 (43–53.6)
Neck circumference (cm)	40.8 (38.8–44.3)	40.0 (38.0–46.0)
Waist/hip ratio	1.0 (1.0–1.1)	1.0 (1.0–1.1)
Smoker (current/ex/never)	2/13/24	0/16/25

**Fig 2:**
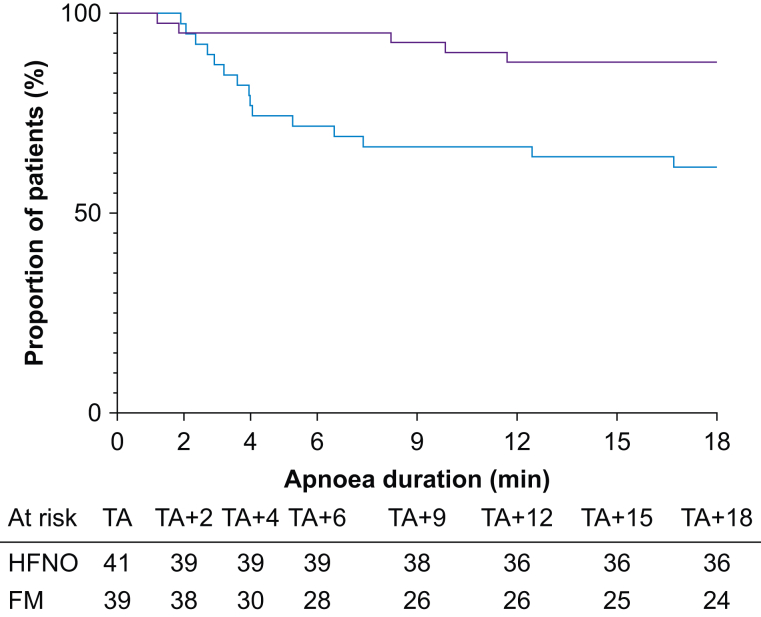
Risk of desaturation during apnoea. Proportion of study participants in HFNO (purple line) and FM (blue line) groups with oxygen saturation >92% during apnoea. Hazard ratio comparing FM and HFNO groups, 0.27 (95% confidence interval, 0.11–0.65; log-rank *P*=0.007). TA, onset of apnoea. TA+*x*, apnoea duration where *x* denotes min after TA; FM, facemask oxygen; HFNO, high-flow nasal oxygen.

Five patients (12%) in the HFNO group and 15 (38%) in the FM group desaturated to 92% within 18 min of apnoea (Fig. 2). The risk of desaturation was significantly lower in the HFNO group than in the FM group (hazard ratio=0.27; 95% CI, 0.11–0.65; log-rank *P*=0.007).

The median time to desaturation was 18 (IQR 18–18) min in the HFNO group and 18 (IQR 4.1–18) min in the FM group. However, these data are heavily right-censored because the majority of patients in both groups reached the 18 min endpoint without desaturating, and therefore statistical analysis (although significant, Mann–Whitney *U P*=0.0068) is of limited value. Of the 20 patients who desaturated, time to desaturation was longer in HFNO group (median=8.2; IQR 1.5–10.8 min; *n*=5) compared with the FM group (median=4.0; IQR 2.7–6.5 min; *n*=15), but this was not statistically significant (Mann–Whitney *U P*=0.933).

Blood gas data were not available for two baseline samples and two post-preoxygenation samples (separate participants) in the HFNO group and three apnoeic samples (TA+2/+4/+6) in one participant in the FM group. Missing samples were all in participants who completed 18 min of apnoea. Two TA+18 samples (both HFNO) showed a marked reduction in *P*a_co2_ (9.4 to 7.8 kPa in one and 9.3 to 7.7 kPa in the other), which were also lower than samples taken after 1 min of ventilation (8.5 kPa in both cases). These samples were excluded from analysis.

Blood gas data are summarised in Table 2. At baseline and after preoxygenation, there was no significant difference in the mean *P*a_o2_ between the two groups.

**Table 2:** *P*a_o2_ and *P*a_co2_ before and during apnoea. Figures shown are mean (standard deviation) arterial oxygen and carbon dioxide tensions (kPa). TA, onset of apnoea; TA+*x*, apnoea duration where *x* denotes min after TA. Figures before apnoea (at baseline breathing air, and at the end of preoxygenation) include data from all participants in the high flow nasal oxygen (HFNO; where data are available, *n*=39) and facemask oxygen (FM, *n*=39) groups. During apnoea, figures include data only from the 60 participants who completed 18 min of apnoea (HFNO, *n*=36; FM, *n*=24). Comparisons were made using the independent *t*-test.

		Baseline	After preoxygenation	TA	TA+2	TA+4	TA+6	TA+9	TA+12	TA+15	TA+18
*P*a_o2_	HFNO	12.8 (2.4)	59.9 (8.1)	55.7 (7.2)	40.4 (8.1)	30.4 (8.1)	28.2 (8.2)	26.7 (7.3)	26.0 (7.2)	24.5 (6.7)	23.6 (6.7)
	FM	12.7 (2.3)	60.2 (9.4)	50.7 (9.9)	35.5 (11.6)	30.5 (11.1)	28.7 (10.2)	27.1 (9.9)	25.2 (9.4)	23.8 (9.1)	22.2 (8.3)
	*P*	0.978	0.978	0.249	0.422	1.000	0.999	0.999	0.999	0.999	0.987
*P*a_co2_	HFNO	5.3 (0.5)	4.5 (0.9)	6.5 (0.9)	7.1 (0.8)	7.7 (0.8)	8.2 (0.8)	9.0 (0.9)	9.6 (0.9)	10.1 (1.0)	10.7 (1.1)
	FM	5.3 (0.5)	5.0 (0.7)	6.6 (0.8)	7.1 (0.7)	7.8 (0.7)	8.3 (0.8)	8.9 (0.8)	9.5 (0.9)	10.0 (1.0)	10.6 (1.1)
	*P*	0.660	0.009	0.996	0.999	0.999	0.999	0.999	0.999	0.996	0.999

To allow direct comparison of the effect of oxygen delivery method on the efficiency of apnoeic oxygenation and ventilation,^3^ analyses of blood gas tension during apnoea were restricted to the 60 patients who reached 18 min without desaturation. There was no significant difference in the mean *P*a_o2_ or *P*a_co2_ at any time point during apnoea.

**Fig 3:**
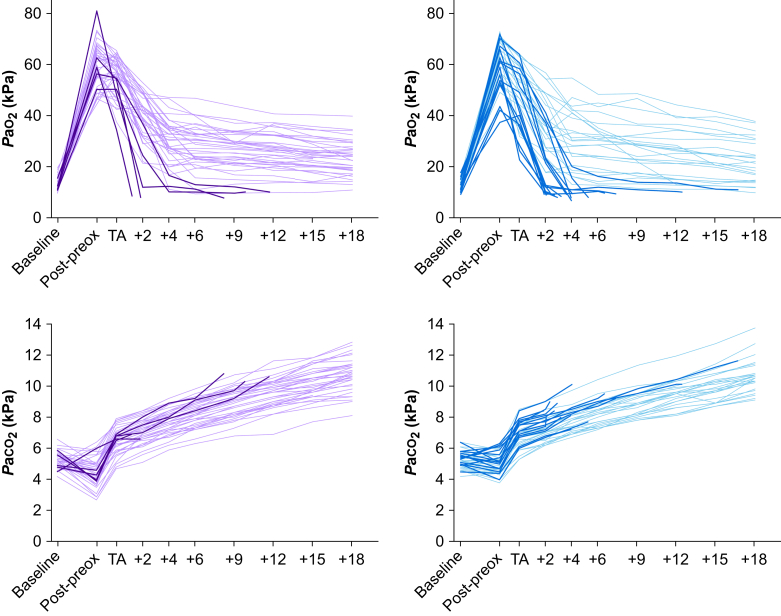
*P*a_o2_ and *P*a_co2_ change over time during apnoea. Change in arterial oxygen and carbon dioxide tension during apnoea. TA, onset of apnoea; TA+*x*, apnoea duration where *x* denotes min after TA. Each line represents an individual participant (purple: high flow nasal oxygen group; blue: facemask group). Light lines represent participants whose oxygen saturation level remained >92% during apnoea throughout the 18 min period. Dark lines represent participants who desaturated to 92% during the study period.

Individual *P*a_o2_ and *P*a_co2_ curves are shown in Fig. 3. Among participants who reached 18 min of apnoea, the mean rate of *P*a_co2_ increase between TA and TA+18 was 0.224 (0.05) kPa min^−1^ in the FM group and 0.232 (0.05) kPa min^−1^ in the HFNO group. This was not significantly different, nor was the rate of increase at any single time point.

## Discussion

The principal finding of this study is that, under these experimental conditions, morbid obesity does not preclude apnoeic oxygenation. Overall, 60 (75%) morbidly obese patients tolerated apnoea for 18 min without desaturation. In the HFNO group, the proportion (36/41, 88%) was similar to that previously reported in non-obese patients^13^ although, in contrast to this study, patients in the non-obese case series were in the supine position.

This is the largest RCT of apnoeic oxygenation in morbidly obese patients to date. Before this, apnoea without desaturation in obese patients had been reported using various oxygen delivery techniques. Using our definition of apnoea onset, the maximum apnoea time in these earlier studies was 10.5 min^14^ and, subsequently, one trial has extended this to 14 min.^15^ There have been no studies of comparable size with the exception of one, studying obese patients, in which the maximum apnoea time was 6 min.^16^

In the FM group, a lower proportion tolerated apnoea without desaturation, suggesting that HFNO improves the efficiency of apnoeic oxygenation. The only directly comparable study is in non-obese older patients which reported similar results.^17^ Our findings align with previous studies which report that both flow rate^18^ and proximity of fresh gas flow to the respiratory epithelium^19^ influence apnoeic oxygenation efficiency.

However, as Fig. 3 shows, there is inter-individual variability in *P*a_o2_ trajectory during apnoea. This is not linear, initially decreasing rapidly before decreasing more slowly. There was substantial variation in both the rate of initial descent and the inflexion point. As Table 2 demonstrates, this variability was similar in HFNO and FM groups. The physiological factors that underlie this variability are not thus far clear.

Oxygen delivery characteristics may mitigate the risk of desaturation^18^ and this study supports this notion. However, the unexpectedly high proportion of patients in the FM group reaching 18 min without desaturation and the wide variability in *P*a_o2_ during apnoea in both groups also suggests that oxygen delivery characteristics are not the primary determinant of whether patients desaturate during apnoea. Indeed, it has been shown that desaturation can occur despite 100% oxygen concentration within the trachea.^20^

This is reinforced by the wide variation seen in results from apnoeic oxygenation studies using similar oxygen delivery techniques. In obese patients, there are no other studies investigating facemask oxygen but three have used HFNO. In one, the mean apnoea time was 261 (77.7) s.^21^ In another, censored at 600 s, the median apnoea time was 600 (IQR 296–600) s.^22^ In the third, censored at 900 s, the median was 537 (IQR 399–808) s.^15^ Notably, in the latter study, there was no difference between apnoea time in patients given high-flow (120 L min^−1^) and low-flow (10 L min^−1^) oxygen. It is not clear why these and our results differ, but oxygen delivery method *per se* does not seem to be the reason. These disparities emphasise the complexity of apnoeic oxygenation as a physiological process and the need for deeper understanding of the mechanisms that underpin it.

In our study, patients were semi-recumbent with a 45° mid-thoracic incline, steeper than other studies in which patients were positioned at 30°,^14^^,^^21^ 25°,^16^ or 20°,^13^ the ‘ramped sniffing’ position,^15^^,^^18^ or supine.^17^ Although there is no clear relationship between apnoea time and position among these studies, comparison is limited by other methodological differences. There is, however, evidence that position affects apnoea time,^23^^,^^24^ and this may have contributed to the apnoea time achieved by patients in our study.

### Apnoeic ventilation

Arterial carbon dioxide tension during apnoea initially increases steeply followed by a more gradual incline.^25^ The underlying physiology is not well understood but contributory factors are arteriovenous admixture, the Haldane effect, and possibly apnoeic ventilation. Evidence for the latter has been derived from comparison of studies reporting *P*a_co2_ increase during apnoea with^25^ (∼0.45 kPa min^−1^) and without^9^^,^^13^^,^^26^^,^^27^ airway obstruction (as low as 0.15 kPa min^−1^). However, these comparisons are confounded by experimental design, particularly the use of proxy measures such as end-tidal carbon dioxide concentration,^3^ which has been shown to be an imprecise marker of *P*a_co2_.^9^

This study provides direct evidence of *P*a_co2_ changes in 60 patients during 18 min of apnoea, the largest such dataset published to date. Overall, the rate of increase was 0.23 (0.05) kPa min^−1^, which suggests that ventilatory exchange is indeed a phenomenon.^3^ This is similar to the rate of increase seen in studies of non-obese patients,^9^^,^^13^^,^^27^ which supports our contention that obesity itself (with appropriate airway management) does not preclude apnoeic gas exchange. Our analysis is limited to those patients who tolerated 18 min of apnoea without arterial oxygen desaturation but our findings suggest that, in this group, the efficiency of this process is not substantially different to that in non-obese patients.

There was no significant difference between HFNO and FM groups in the rate of *P*a_co2_ increase during apnoea. This challenges the notion that HFNO delivery improves efficiency of ventilatory exchange^10^^,^^28^ and, correspondingly, that enhanced carbon dioxide clearance is responsible for any increase in efficiency of apnoeic oxygenation when high gas flows are used.^29^

It has been hypothesised that apnoeic carbon dioxide clearance may become more efficient as alveolar concentration increases.^9^ We did not observe a ‘plateau’ where *P*a_co2_ stopped increasing. This may require a longer period of apnoea but it is questionable whether it is clinically relevant, particularly in the context of mounting acidaemia.^30^

### Strengths and limitations

This physiological study was conducted in a safe environment by experts in bariatric anaesthesia. These conditions allowed the safe collection of an unprecedented dataset. In comparison with earlier case series, this was a systematic study which minimised risk of confounding. This study was clinically relevant: airway management can be challenging and time-pressured in obese patients owing to rapid desaturation. However, our findings may not be transferable to other populations.

Our primary outcome, in common with most other studies of apnoeic oxygenation, was time to desaturation. In previous studies,^12^^,^^14^^,^^31^ the majority of patients in the intervention groups reached the census point (the maximum duration of apnoea allowed by the study design) without desaturation. This limits the clinical utility of this outcome measure as it is predominantly determined by the choice of census point rather than the physiological performance of the patients. In response, we increased the maximum apnoea time to 18 min. Despite this and unexpectedly, the majority of patients reached this census point without desaturation. This was also a limitation of other studies performed around the same time as ours, which also used longer apnoea periods.^18^^,^^20^^,^^32^

Time to desaturation as an outcome is therefore sensitive to study design and it does not describe the risk of desaturation during apnoea. In retrospect it is clear that desaturation risk, as used by a recent study in high-risk patients undergoing endoscopy,^33^ would have been a better primary outcome. A strength of this study is the temporal resolution of the blood gas dataset, which illustrates the fallibility of time to desaturation as an outcome in apnoeic oxygenation studies (Fig. 3).

The majority of patients who desaturated in this study did so early in apnoea. In these cases the apnoea time was less than 5 min, comparable with time to desaturation in obese patients who were not given supplemental oxygen during apnoea.^14^^,^^34^ This pattern, an apparent dichotomy between patients who do and do not ‘tolerate’ apnoea, was reported as early as 1973^35^ and has been replicated in studies in both obese^14^^,^^18^ and non-obese patients.^17^

Clinically, this is an important finding: the implication is that for some patients – whatever the delivery method – supplying oxygen does not greatly increase the duration of apnoea without desaturation. Indeed, it is questionable whether the notion of ‘safe apnoea time’ is valid.

A technical limitation to this study relates to blood sampling method: blood gas samples were not processed contemporaneously but were refrigerated until completion of an individual patient study run. However, samples were analysed sequentially and each analysis took approximately 2 min, which approximately matches the sampling time difference. Two blood gas samples taken at intubation showed a noticeably lower *P*a_co2_ (7.8 and 7.7 kPa) compared with previous (9.4 and 9.3 kPa, respectively) and subsequent samples (both 8.5 kPa after 1 min of mechanical ventilation). In one case, other biochemical markers were consistent with saline contamination. In the other, the reason for the decrease is not clear. These discrepancies were obvious and have been excluded but other sampling discrepancies may have been missed. However, the fact that all of the other blood gas data points followed coherent trajectories (Fig. 3) suggests that this was not a significant methodological concern.

Although we did not monitor neuromuscular block, the protocolised dose of rocuronium (1 mg kg^−1^) should have been adequate to prevent diaphragmatic movement.^36^^,^^37^ The majority of patients received additional rocuronium during apnoea, including 54 of the 60 patients who reached 18 min without desaturation, which suggests that this was due to apnoeic oxygenation rather than imperceptible diaphragmatic movement.

In contrast to some studies,^12^^,^^14^^,^^31^ no direct visualisation of the airway was used throughout apnoea. However, an oropharyngeal airway and jaw thrust were used and a check that manual ventilation was possible at the beginning of apnoea confirmed the airway was patent at this point. Theoretically, operator fatigue during prolonged jaw thrust may have led to airway compromise after this but most patients who desaturated did so within the first 5 min, suggesting that this did not materially alter results.

### Clinical relevance/application

Although this study demonstrates that apnoeic oxygenation is possible in morbidly obese patients, it was conducted under experimental conditions by bariatric anaesthetists with the experience and resources to manage any complication. Patients were screened to exclude conditions that might put them at predictable risk of complications during apnoea or airway management. This was primarily a physiological study and the experimental conditions do not translate to clinical conditions such as ‘tubeless’ airway surgery.

Clinically, this study highlights two important, in some ways contradictory, precepts: first, a note of caution that patients may desaturate rapidly during apnoea despite optimal preoxygenation and an adequate oxygen supply. The factors that determine this risk – and which may be present also in non-obese patients – warrant further investigation. Second, the majority, including those in the FM group, tolerated apnoea without desaturation for a duration that substantially exceeds the normal duration of perioperative airway management. This suggests that apnoea is tolerable in most patients using a standard anaesthetic facemask under certain conditions – notably, adequate preoxygenation, head-up position, intravenous anaesthetic delivery, haemodynamic stability, and assiduous attention to mask seal and airway patency.

The safety of this manoeuvre presupposes the ability to facemask ventilate in the event of desaturation. Potential advantages of minimising facemask ventilation relate to the risk of gastric insufflation, which may increase the risk of regurgitation and aspiration of gastric contents, compromise laparoscopy and ventilation, and increase the risk of postoperative nausea and vomiting. Recently, the risk of aerosol generation during facemask ventilation has been a concern, and this technique may be one element among many in the management of this risk.

## Authors' contributions

Study concept and design: JC, AL, SJB, AK

Data acquisition: JC, JSW, TW, RS, AK, RK, AA, SP

Data analysis: JSW, JC, TW, RS

Data interpretation: JC, JSW, TW, RS, SJB

Writing of the initial manuscript draft: JSW, TW, JC, RS

Critical revision: JSW, JC, SJB, TW, RS, AK, AL, RK

## Declarations of interest

JC has received payments and travel funding from Fisher & Paykel Healthcare, Auckland, New Zealand (Fisher & Paykel) for both UK and overseas lectures. He continues to advise Fisher & Paykel on future developments relating to delivery of humidified oxygen. TW has previously received travel funding from Fisher & Paykel. The other authors have no relevant interests to declare.

## Funding

The National Institute for Health Research 10.13039/501100013342Imperial Biomedical Research Centre provided infrastructure support. Fisher & Paykel provided high-flow nasal oxygen equipment and disposables, but were not involved with design, data collection, analysis, or interpretation of the study and had no role in the preparation of the manuscript or the decision to submit the manuscript for publication.
